# Ultra-bright *γ*-ray emission and dense positron production from two laser-driven colliding foils

**DOI:** 10.1038/s41598-017-17605-6

**Published:** 2017-12-11

**Authors:** Han-Zhen Li, Tong-Pu Yu, Jin-Jin Liu, Yan Yin, Xing-Long Zhu, Remi Capdessus, Francesco Pegoraro, Zheng-Ming Sheng, Paul McKenna, Fu-Qiu Shao

**Affiliations:** 1 College of Science, National University of Defense Technology, Changsha, 410073 China; 2 SUPA, Department of Physics, University of Strathclyde, Glasgow, G4 0NG UK; 3 Institute of Applied Physics and Computational Mathematics, Beijing, 100094 China; 4 Collaborative Innovation Center of IFSA (CICIFSA), Key Laboratory for Laser Plasmas (MoE) and School of Physics and Astronomy, Shanghai Jiao Tong University, Shanghai, 200240 China; 5 Department of Physics Enrico Fermi, University of Pisa, and CNR/INO, Pisa, 56122 Italy; 6 Tsung-Dao Lee Institute, Shanghai, 200240 China; 7 Cockcroft Institute, Sci-Tech Daresbury, Cheshire, WA4 4AD UK

## Abstract

Matter can be transferred into energy and the opposite transformation is also possible by use of high-power lasers. A laser pulse in plasma can convert its energy into *γ*-rays and then *e* ^−^ *e* ^+^ pairs via the multi-photon Breit-Wheeler process. Production of dense positrons at GeV energies is very challenging since extremely high laser intensity ~10^24^ Wcm^−2^ is required. Here we propose an all-optical scheme for ultra-bright *γ*-ray emission and dense positron production with lasers at intensity of 10^22–23^ Wcm^−2^. By irradiating two colliding elliptically-polarized lasers onto two diamondlike carbon foils, electrons in the focal region of one foil are rapidly accelerated by the laser radiation pressure and interact with the other intense laser pulse which penetrates through the second foil due to relativistically induced foil transparency. This symmetric configuration enables efficient Compton back-scattering and results in ultra-bright *γ*-photon emission with brightness of ~10^25^ photons/s/mm^2^/mrad^2^/0.1%BW at 15 MeV and intensity of 5 × 10^23^ Wcm^−2^. Our first three-dimensional simulation with quantum-electrodynamics incorporated shows that a GeV positron beam with density of 2.5 × 10^22^ cm^−3^ and flux of 1.6 × 10^10^/shot is achieved. Collective effects of the pair plasma may be also triggered, offering a window on investigating laboratory astrophysics at PW laser facilities.

## Introduction

The rapid development of laser technologies promises substantial growth of peak laser intensities. As of today, laser intensity of 10^22^ Wcm^−2^ has been demonstrated^1^ and several laser facilities such ELI^2^ and APOLLON^3^, towards focused intensities above 10^23^ Wcm^−2^ are under construction. The electron dynamics in such intense laser fields approaches the radiation-reaction dominated quantum-electrodynamics (QED) regime^4–6^. Bright *γ*-rays emission, *e* ^−^ *e* ^+^ pairs production, QED-cascade as well as energetic particles acceleration are highly coupled, forming an *e* ^−^ *e* ^+^-*γ* plasma^7,8^. This is a totally unexplored research area, which opens up new avenues in high energy density physics, particle and nuclear physics, and high energy astrophysics such as *γ*-ray bursts, pulsars, and active galactic nuclei in laboratories^9–12^.

Breit-Wheeler (BW) pair production is a physical process in which an *e* ^−^ *e* ^+^ pair is created in the collision of two photons^13^. At extremely high laser intensities, an important mechanism for *e* ^−^ *e* ^+^ pair production is the multi-photon BW process^7^, which enables the laser energy to convert into copious *e* ^−^ *e* ^+^ pairs via photon-photon annihilation ($\gamma +n\hslash {\omega }_{l}\to {e}^{-}+{e}^{+}$). Here, *ω*_*l*_ is the laser photon frequency. Generally, the low energy photons are laser photons or thermal emission^14^, while high energy *γ*-photons result from accompanying radiation by relativistic electrons propagating in medium (bremsstrahlung^14–17^), colliding with laser pulses (Compton back-scattering^6,18–21^), or oscillating in intense electric fields (synchrotron/betatron radiation and skin-depth emission^22–27^). At laser intensities of ~10^23–24^ Wcm^−2^, several schemes are recently proposed to generate bright *γ*-rays and dense *e* ^−^ *e* ^+^ pairs in the laser-driven QED regime^6,7,20–33^. Broadly, these attempts can be classified into three main categories: a super-intense laser or lasers interaction with (i) a solid Al target^7,23,24,28^, (ii) near-critical-density (NCD) plasmas^20,29^, or (iii) an energetic electron beam (or single electron^31^) driven by laser wakefield^6,21,32^ in gas plasmas or by a conventional accelerator^19,33^. Among them, significant attention has been paid to the first category for overdense *e* ^−^ *e* ^+^ pair production, which may trigger collective effects or ‘medium-like behavior’, e.g., Debye Shielding, required for modeling astrophysical phenomena in laboratories. However, these studies are limited to either one-dimensional (1D) theory or 2D particle-in-cell (PIC) simulations, and the inherent deficiency, i.e, high laser reflection by the immobile target, results in relatively low laser energy conversion. A full understanding of QED physics during the laser-foil interaction and improving the laser energy conversion in real 3D configuration become pivotal and pressing.

**Figure 1:**
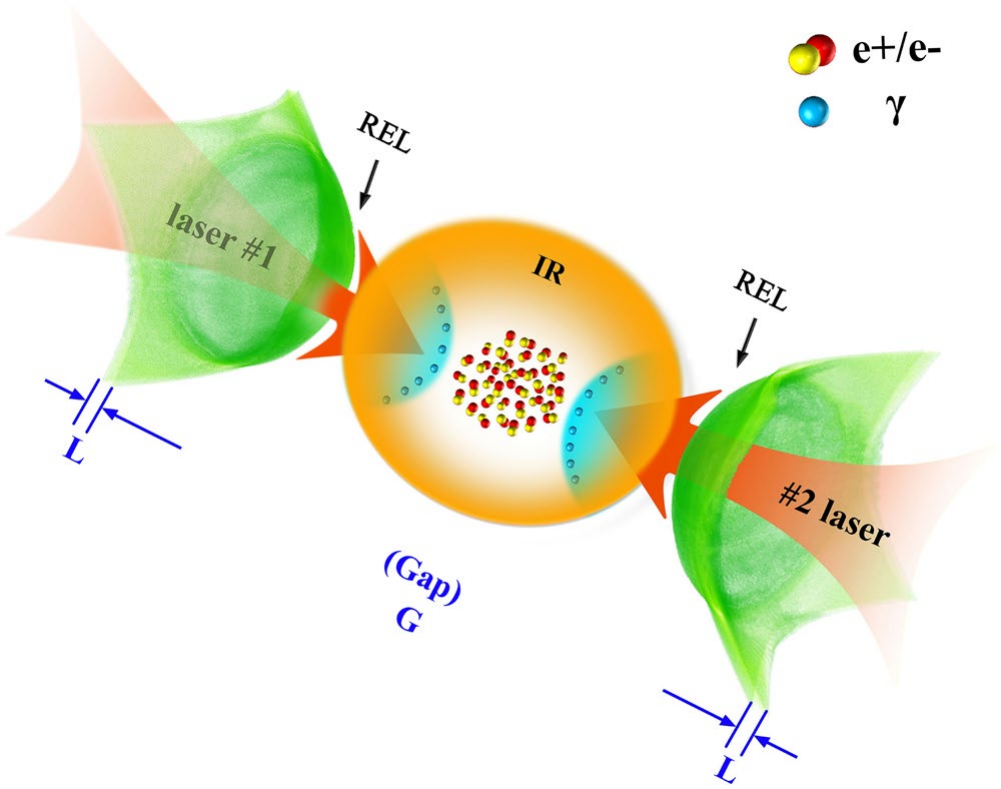
Schematic diagram of ultra-bright *γ*-ray emission and dense *e* ^−^ *e* ^+^ pair production by counter-propagating lasers irradiating two diamondlike carbon (DLC) foils.

At laser intensities of ~10^22,23^ Wcm^−2^, much less attention has been given to *e* ^−^ *e* ^+^ pair production due to the small flux of the high energy *γ*-photon emission. Here, we propose a novel scheme for ultra-bight *γ*-ray emission and dense positron production at achievable laser intensities in the foreseeable future. As shown in Fig. 1, two elliptically-polarized (EP) lasers are incident onto two diamondlike carbon (DLC) foils. The electrons in the center area of foils (i.e. the region of the laser focal spot) are rapidly accelerated to high velocities by the laser pressure and form overdense relativistic electron layers (RELs). Due to strong electron heating and foil expansion, relativistic transparency of both foils occurs under certain conditions, such that the laser penetrates through one foil and collide with the counter-propagating compressed layer of electrons accelerated from the other foil. This symmetric configuration enables efficient Compton back-scattering and increases equivalent quantum invariants, resulting in ultra-bright *γ*-ray emission with an unprecedented peak brightness and dense GeV positron beam production. The laser intensity required is within the capabilities of future multi-PW laser facilities, paving the way to potential applications in nuclear and particle physics for fundamental research, laboratory study of astrophysics, medical imaging and material science^4,5,9–11^.

## Results

### Radiation pressure acceleration of both foils by EP laser pulses

There has been particular interest in radiation pressure acceleration (RPA) of ultra-thin foils using circularly-polarized (CP) laser pulses, as they are capable of suppressing fast electron generation and resulting in quasi-monoenergetic ion beams^34–42^. In experiments, however, EP laser pulses are far more general because it is usually very difficult to get pure CP pulses at extreme intensities. When such an EP pulse irradiates a thin foil, both the characteristics of a CP and linearly polarized (LP) pulse are present. On the one hand, like a CP pulse, the laser radiation pressure pushes the thin foil forward as a whole; on the other hand, using EP laser pulses can suppress the transverse instabilities, which is beneficial to the foil acceleration^43^. In our scheme, two colliding EP laser pulses with a polarization ratio as predicted in ref.^43^ are employed. Meanwhile, DLC foils are used instead of common metals like Au and Al employed previously^15–17,20,22–24^. It is shown that the DLC foils can offer exceptionally high tensile strength, hardness, and heat resistance, making them ideally suited materials for self-supporting targets in experiments^36,44^; unlike immobile metal targets, the foil dynamics in our proposed scheme is coupled with the *γ*-ray emission, enabling efficient laser energy conversion to electrons, and then *γ*-photons and positrons. By using such low-Z thin foils, *e* ^−^ *e* ^+^ pair production is dominated by the multi-photon BW process, while alternative one, i.e., Bethe-Heitler (BH) process^45,46^ related with high-Z atoms and thick targets can be ignored (see Methods).

**Figure 2:**
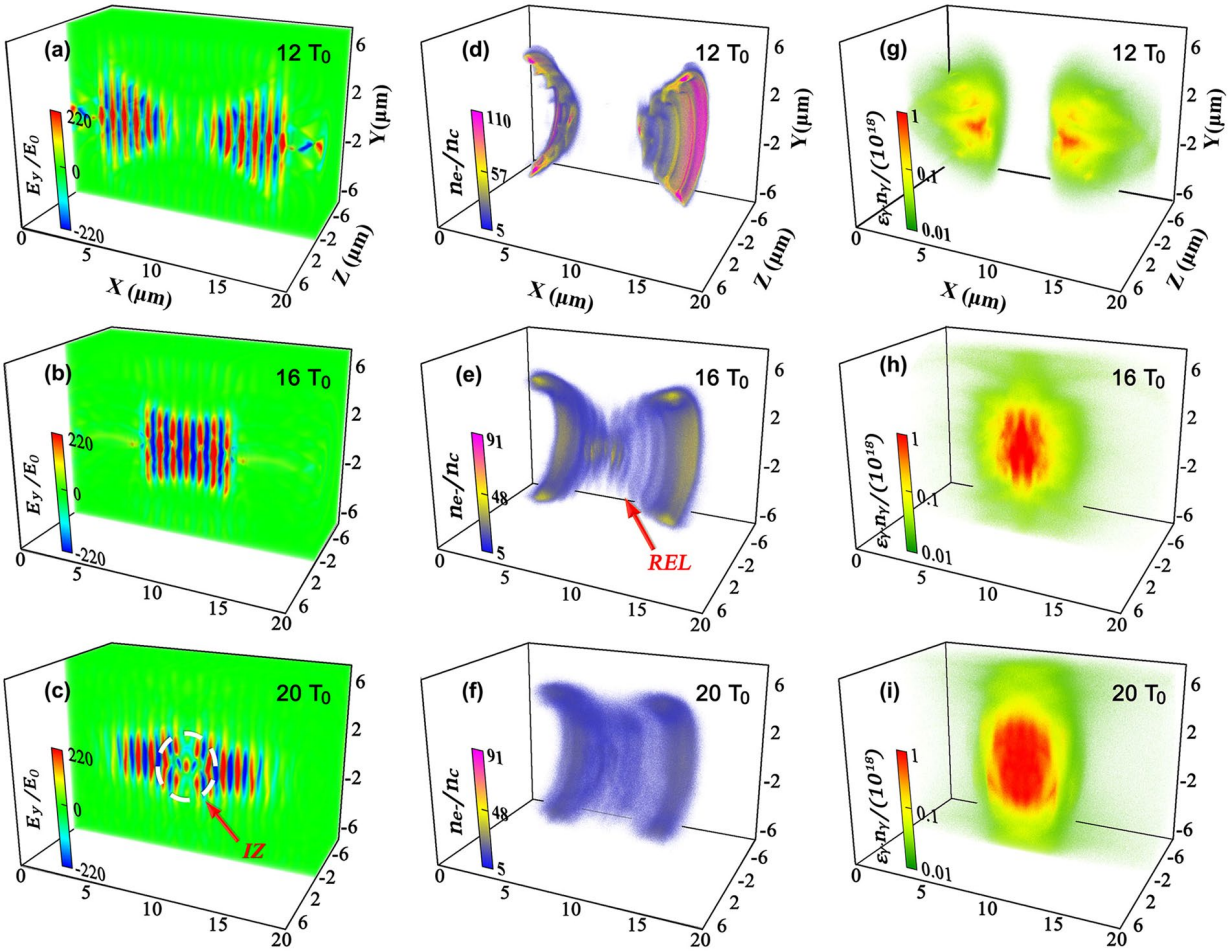
Distributions of the transverse electric field (**a**–**c**), electron density (**d**–**f**), and photon energy density (**g**–**i**) at *t* = 12*T*_0_, 16 *T*_0_ and 20 *T*_0_, respectively. Here, ${E}_{0}={m}_{e}c{\omega }_{0}/e=3.2\times {10}^{12}$ Vm^−1^. The white dashed circle in (**c**) refers to the interaction zone (IZ) and the red arrow in (**e**) points to the relativistic electron layers (RELs).

Due to the surge of abundant secondary particles during the laser-target interaction, previous studies were usually limited to 1D or 2D simulations as 3D simulations are extremely computational expensive. Here we perform the first 3D simulation using QED-PIC code EPOCH^47,48^ (see Methods) to demonstrate ultra-bright *γ*-ray emission and dense positron production from laser-driven DLC foils. Fig. 2 presents the main simulation results. During the foil acceleration at the initial stage, carbon ions and protons are separated from each other with protons at the leading edge of the carbon ion front, due to their higher ion charge-to-mass ratio^39,49,50^. Note that ${a}_{0}=\sqrt{{a}_{y}^{2}+{a}_{z}^{2}}\mathrm{ < 2}\Xi \equiv 2({n}_{{e}^{-}}/{n}_{c})(\pi L/{\lambda }_{0})$, which is not strong enough to blow all electrons out of the foil. Thus a depletion region with a length of *x*_*d*_ for electrons forms in the foil. This generates strong charge separation fields, which in turn pull ions forward. At ${x}_{l}=D+{x}_{d}$ and ${x}_{r}=D+G-{x}_{d}$ (see Methods) on both sides, the electron density has a maximal value in the *x*-axis, which is high enough to prevent the laser pulse from penetrating through the foil. During this initial stage, both foils almost keep the structure intact and the degree of electron heating is not significant in such a short time duration. Due to the transversely Gaussian distribution of the laser intensity, both foils are distorted and a cone structure forms at 12*T*_0_, as shown in Fig. 2(a) and (d). Some electrons within the skin-depth of the foil’s inner wall are pulled out by the traveling laser pulses and interact with the reflected waves^22^. However, both the electron energy and number are relatively small and the reflected waves are much weaker due to Doppler red shifting, so that only a few *γ*-photons are emitted and no positrons are produced at this stage (see Fig. 2(g)). This is markedly different from the cases in refs^22–24^, where ~10^24^ Wcm^−2^ laser pulses are adopted and *e* ^−^ *e* ^+^ pairs can be produced during the hole-boring RPA.

With the foil deformation and thermal expansion, the electron density along the laser axis decreases, while its energy increases. At the same time, Rayleigh-Taylor-like instability (RTI) grows quickly^51–54^. The physics associated with the foil acceleration is complex in full dimensions here, however, we may get deeper insights from the the 1D light-sail (LS) mode^37–40^. Taking into account only the center foil with a size of the laser focal spot where is of interest for the multi-photon BW process, the foil dynamics is well described by the motion equation $d(\gamma \beta )/dt=$ $\mathrm{(2}I/\rho {c}^{2})R(\omega ^{\prime} \mathrm{)(1}-\beta \mathrm{)/(1}+\beta )$, where $I=\mathrm{(1}+{\zeta }^{2})c{E}_{y}^{2}\mathrm{/8}\pi$ is the laser intensity, $\zeta ={a}_{z}/{a}_{y}=0.65$, *E*_*y*_ is the laser electric field in the *y*-axis, $R(\omega ^{\prime} )$ is the reflectivity in the rest frame of the foil with $\omega ^{\prime} ={\omega }_{0}\sqrt{\mathrm{(1}-\beta \mathrm{)/(1}+\beta )}$, ${\omega }_{0}=2\pi /{T}_{0}$, *β* is the foil velocity, $\gamma =\mathrm{1/}\sqrt{1-{\beta }^{2}}$, and $\rho ={\sum }_{i}{m}_{i}{n}_{i}L$ is the foil areal mass density with ${m}_{i}={Z}_{i}{m}_{p}$, *n*_*i*_ and *Z*_*i*_ being the ion density and mass number, respectively. Since we are interested in only the carbon ion front, we may assume perfect reflection of the incident laser pulse by the foil. Simplifying the motion equation above, we thus get

1 $$\frac{d\beta }{dt}=\frac{(1+{\zeta }^{2}){E}_{y}^{2}}{4\pi \rho c}{\mathrm{(1}-\beta )}^{2}\sqrt{1-{\beta }^{2}},$$

**Figure 3:**
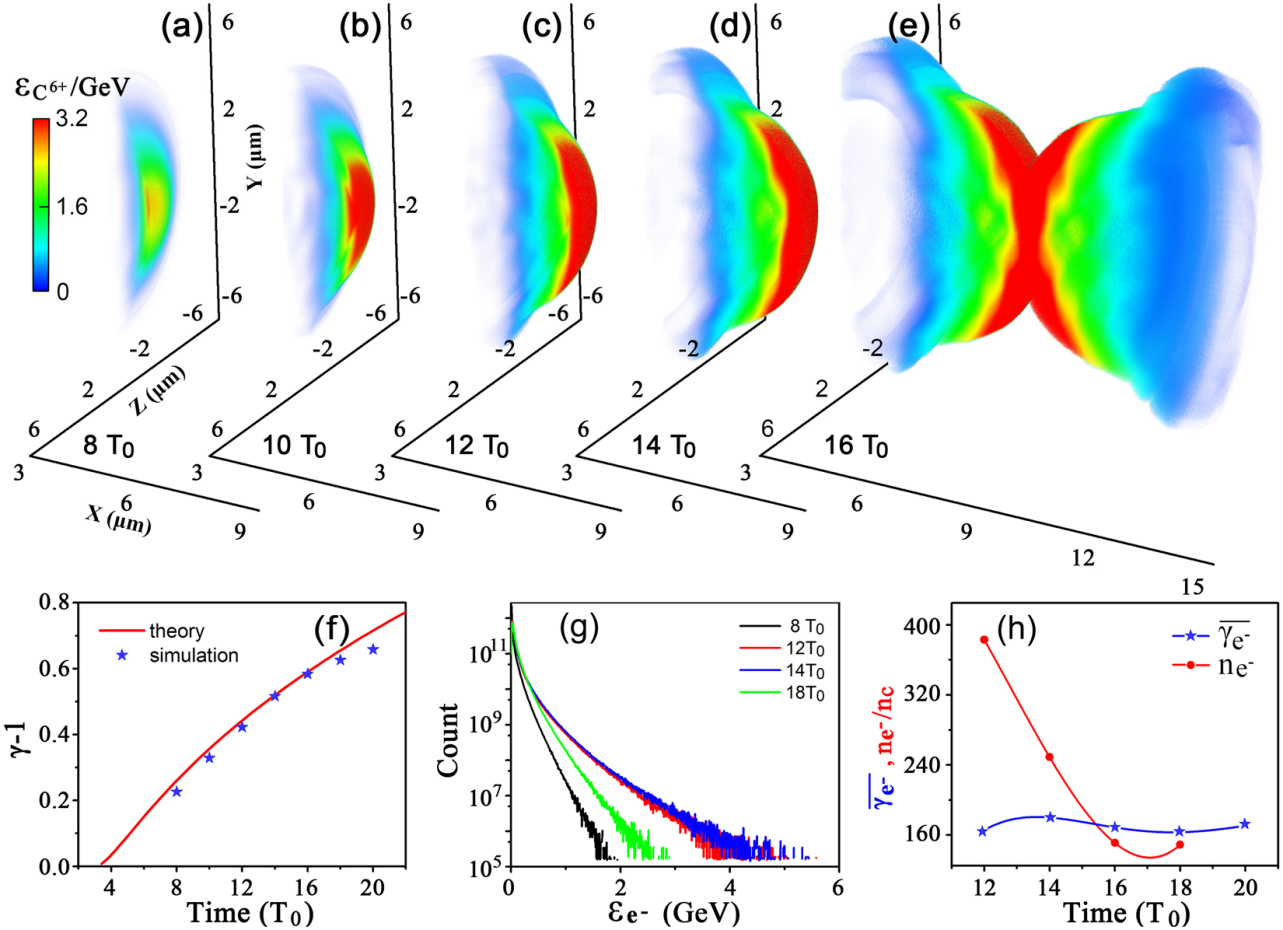
Carbon ion energy distributions (**a**–**e**), energy evolution of the carbon ion front (**f**), electron energy spectrum (**g**), and evolution of $\overline{{\gamma }_{{e}^{-}}}$ and the maximum electron density *n*_*e*_ (**h**). The solid curves in (**h**) serve as guides to the eye.

Introducing a new parameter $\kappa =(1+{\zeta }^{2}){E}_{y}^{2}\mathrm{/4}\pi \rho c$ and solving Eq. (1) analytically, we obtain the *γ* evolution of the foil in the 1D LS mode as

2 $$\gamma =\frac{1}{\sqrt{1-{\mathrm{(1}-{[\nu (t)+\upsilon (t)]}^{\mathrm{1/3}}-{[\nu (t)-\upsilon (t)]}^{\mathrm{1/3}})}^{2}}},$$

where $\nu (t)=1/[1+\varsigma {(t)}^{2}]$, $\upsilon (t)=\varsigma (t)/{[1+\varsigma {(t)}^{2}]}^{\mathrm{3/2}}$ and $\varsigma (t)=3\kappa t+2$ with $\kappa =\mathrm{(1}+{\zeta }^{2}){a}_{y}^{2}{m}_{p}{n}_{c}{\lambda }_{0}/\rho {T}_{0}$ (SI). Fig. 3(a–e) present the carbon ion energy distribution in space and Fig. 3(f) shows the evolution of the maximal carbon ion energy per nuclei. Here we only consider the ion acceleration in a smaller size of the same order of the laser spot. We see the center part of both foils already collide with each other at 16*T*_0_, before which the simulation results agree well with the predictions of Eq. (2), demonstrating the dominance of RPA ion acceleration in the region of the laser focal spot.

For the laser intensity under consideration, the radiation friction force does not strongly affect the ion acceleration^55,56^. By use of DLC foils, the electron acceleration becomes inefficient because of the heavier carbon ions. The electrons average energy is lower, leading to a much smaller ration of the friction force to the Lorenz force, ${F}_{R}/{F}_{L}=\mathrm{(2/3)}{k}_{0}{r}_{e}{\gamma }_{{e}^{-}}^{2}{\beta }_{{e}^{-}}{a}_{0}$. Here, ${k}_{0}=2\pi /{\lambda }_{0}$, *r*_*e*_ is the classical electron radius, and ${\beta }_{{e}^{-}}$ is the electron velocity. It is shown that the friction force only equals to the Lorenz force when the threshold *a*_0_ ~ 350 is met (assuming $\overline{{\gamma }_{{e}^{-}}}=500$ in the center zone of the simulation box). Meanwhile, the radiation reaction for electron propagation in the direction of a propagating wave is much smaller than that produced due to counterpropagation^57^. This further mitigates the radiation reaction effect on the electron dynamics. The agreement of the results from the 1D RPA model with the simulation results in Fig. 3(f) justifies the accuracy of the 1D model without consideration of radiation reaction for the ion acceleration in this stage. Although the radiation reaction has limited impact on the ion acceleration, the electron dynamics is affected, which becomes more important at later stages and results in the foil expanding in both longitudinal and transverse directions.

### Relativistically induced transparency of both foils

As the foil undergoes significant deformation, the laser pulses are further focused in the inner bent surfaces and the laser intensity along the axis increases, so that a large number of electrons escape the foils at *t* = 14*T*_0_. The accompanying electron heating becomes important and the RTI develops quickly, leading to the fast expansion of foils along both the longitudinal and transverse directions. However, the later development of the RTI is favorable here, which enables the foil slightly transparent for the incident pulse at *t* = 13*T*_0_ and completely transparent before *t* = 15*T*_0_. Finally, most of the laser waves penetrate through the foil, propagate along the laser axis, and collide with the relativistic electrons from the other side, as is seen in Fig. 2(b) and (e).

Figure 3(g) shows the evolution of electron energy spectrum. At *t* = 14*T*_0_, most high energy electrons with ${\gamma }_{{e}^{-}} > 2000$ originate from the formed RELs as pointed out by the red arrow in Fig. 2(e). The cutoff energy is up to 5 GeV, but it decreases to 3 GeV at *t* = 18*T*_0_. Meanwhile, the corresponding average electron energy also reduces from 92 MeV to 83 MeV. In order to explore the underlying physics, we first consider the time evolution of average electron energy ($\overline{{\gamma }_{{e}^{-}}}$) and the maximum density (${n}_{{e}^{-}}$) in the center area as shown in Fig. 3(h). We see the electron density decreases with the time, gets closer and closer to the $\overline{{\gamma }_{{e}^{-}}}$ curve and finally intersects the curve at about 15*T*_0_. This intersection point refers to the time instant when the laser pulse penetrates through the remainder of the foil in the center area, since the relativistically corrected plasma frequency ${\omega }_{pe}^{R}={\omega }_{l}\sqrt{{n}_{{e}^{-}}/{\gamma }_{{e}^{-}}{n}_{c}}$ becomes smaller than the laser frequency^58–60^. The electron temperature is related to the RTI and also the radiation reaction effect, whose increase may result in the foil expanding in both longitudinal and transverse directions. We thus consider two limit mechanisms, that of a transversely expanding thin foil and of a heated plasma that expands longitudinally. Firstly, we can interpret the transparency occurrence of a thin transversely expanding foil by Bulanov’s condition^58,59^. For a laser pulse with ${a}_{0} > {\rm{\Xi }} > 1$, a foil is transparent to relativistically strong laser radiation if ${a}_{0} \sim ({\varepsilon }_{p}/{{\rm{\Lambda }}}_{y}{{\rm{\Lambda }}}_{z})\sqrt{\mathrm{(1}+\beta \mathrm{)/(1}-\beta )}$ is satisfied. Here ${\varepsilon }_{p}\mathrm{=2}\pi {n}_{{e}^{-}}L{e}^{2}/{m}_{e}{\omega }_{0}c$ and ${{\rm{\Lambda }}}_{y,z}$ is the shell transverse expansion factor. We can rewrite the above condition as

3 $$\sqrt{1+{\zeta }^{2}}{a}_{y} \sim \frac{{\rm{\Xi }}}{{{\rm{\Lambda }}}_{y}{{\rm{\Lambda }}}_{z}}\sqrt{\frac{1+\beta }{1-\beta }},$$

From our simulations, it is shown that the surface stretching factor scales as ${{\rm{\Lambda }}}_{y}{{\rm{\Lambda }}}_{z} \sim 2$ in two laser periods, which is compatible with the traverse expansion velocities of the order of *c*/3. Thus we get a reduction of the opacity by a factor more than 2 between 13*T*_0_ and 15*T*_0_. It is sufficient to make the foil locally transparent. As time progresses, the whole foil becomes transparent to the incident laser pulse. On the other hand, considering the significant electron heating and foil longitudinally expanding in space, we may assume the foil to be a bulk plasma and hole-boring RPA may also occur at the later stage^61^. This can be roughly modeled as a semi infinite plasma, since the expanded foil’s width is larger than the electron skin depth during the acceleration. For an initial overdense plasma foil with a sharp boundary, the pondermotive force pushes electrons into the plasma, which creates strong peaking of the plasma electron density and results in significant enhancement of the laser threshold of penetration. Here, the laser threshold intensity *a*_*d*_ at the electron depletion boundary ${x}_{l}=D+{x}_{d}$ and ${x}_{r}=D+G-{x}_{d}$ on both sides satisfies the relation^62,63^ $3{n}_{{e}^{-}}\mathrm{/2}{n}_{c}\sqrt{1+{a}_{d}^{2}}={n}_{{e}^{-}}/{n}_{c}+{a}_{d}^{2}$. When the incident laser amplitude approaches its maximum value, the corresponding boundary amplitude *a*_*d*,*max*_ has exactly the same value at which the electron density vanishes. Considering ${a}_{d}\gg {n}_{{e}^{-}}/{n}_{c}\gg 1$ here, we obtain the electron density threshold for the foil transparency by ${n}_{th}/{n}_{c}=2[3+\sqrt{9\sqrt{6}{a}_{0}-12}]/9$, which approximates as ${n}_{th} \sim 2\sqrt{\sqrt{6}{a}_{0}}/3{n}_{c}$. In our scheme, due to the multi-dimensional effects such as the foil expanding, laser focusing, electron heating, and the RTI, partial foil transparency is first induced at locations with the electron density smaller than the threshold ${n}_{th} \sim 20{n}_{c}$. This agrees well with simulation results in Fig. 2(e) and (f). In spite of two different models, both predict the occurrence of the foil transparency at about *t* = 15*T*_0_.

### Enhanced *γ*-photon emission and dense *e*^−^*e*^+^ pair production

In the QED regime, the probability rate for photon emission is characterized by the quantum invariant^4^ ${\chi }_{{e}^{-}}=\mathrm{(1/}{a}_{S})\sqrt{{({\varepsilon }_{{e}^{-}}{\bf{E}}+{{\bf{p}}}_{{e}^{-}}\times {\bf{B}})}^{2}-{({{\bf{p}}}_{{e}^{-}}\cdot {\bf{E}})}^{2}}$, where ${a}_{S}=e{E}_{S}/{m}_{e}c{\omega }_{0}={m}_{e}{c}^{2}/\hslash {\omega }_{0}$ is the normalized QED critical field^64^, ${E}_{S}={m}_{e}^{2}{c}^{3}/(\hslash e)=1.32\times {10}^{18}$ Vm^−1^, ${\varepsilon }_{{e}^{-}}={\gamma }_{{e}^{-}}{m}_{e}{c}^{2}$ is the electron energy and ${p}_{{e}^{-}}={\gamma }_{{e}^{-}}{m}_{e}c$ is the electron momentum, **E** and **B** are the electromagnetic field experienced by electrons. For an electron co-propagating with the laser pulse, this results in ${\chi }_{{e}^{-}}\underline{ \sim }0$ and there are almost no high energy *γ*-photons emitted. On the contrary, when the electron counter-propagates with the pulse, one gets ${\chi }_{{e}^{-}}\simeq 2{\gamma }_{{e}^{-}}E/{E}_{s}$ and efficient *γ*-photon emission is achieved if ${\chi }_{{e}^{-}}\ge 1$ ^28^. As the high energy *γ*-photons collide with the low energy ones, it has a probability for *e* ^−^ *e* ^+^ pair production via the multi-photon BW process, which is determined by another quantum parameter^4^, ${\chi }_{\gamma }=(1/{a}_{S})\sqrt{{({\varepsilon }_{\gamma }{\bf{E}}+{{\bf{p}}}_{\gamma }\times {\bf{B}})}^{2}-{({{\bf{p}}}_{\gamma }\cdot {\bf{E}})}^{2}}\simeq (2\hslash {\omega }_{\gamma }/{m}_{e}{c}^{2})E/{E}_{s}$. Here ${\varepsilon }_{\gamma }=\hslash {\omega }_{\gamma }$, ${p}_{\gamma }=\hslash {\omega }_{\gamma }/c$, and *ω*_*γ*_ the photon frequency. One sees that the pair production severely hinges on the local photon energy $\hslash {\omega }_{\gamma }$ and the electric field *E* in the interaction zone. As *χ*_*γ*_ approximates to the unity, QED-cascade induced avalanche-like pair production occurs^31^.

**Figure 4:**
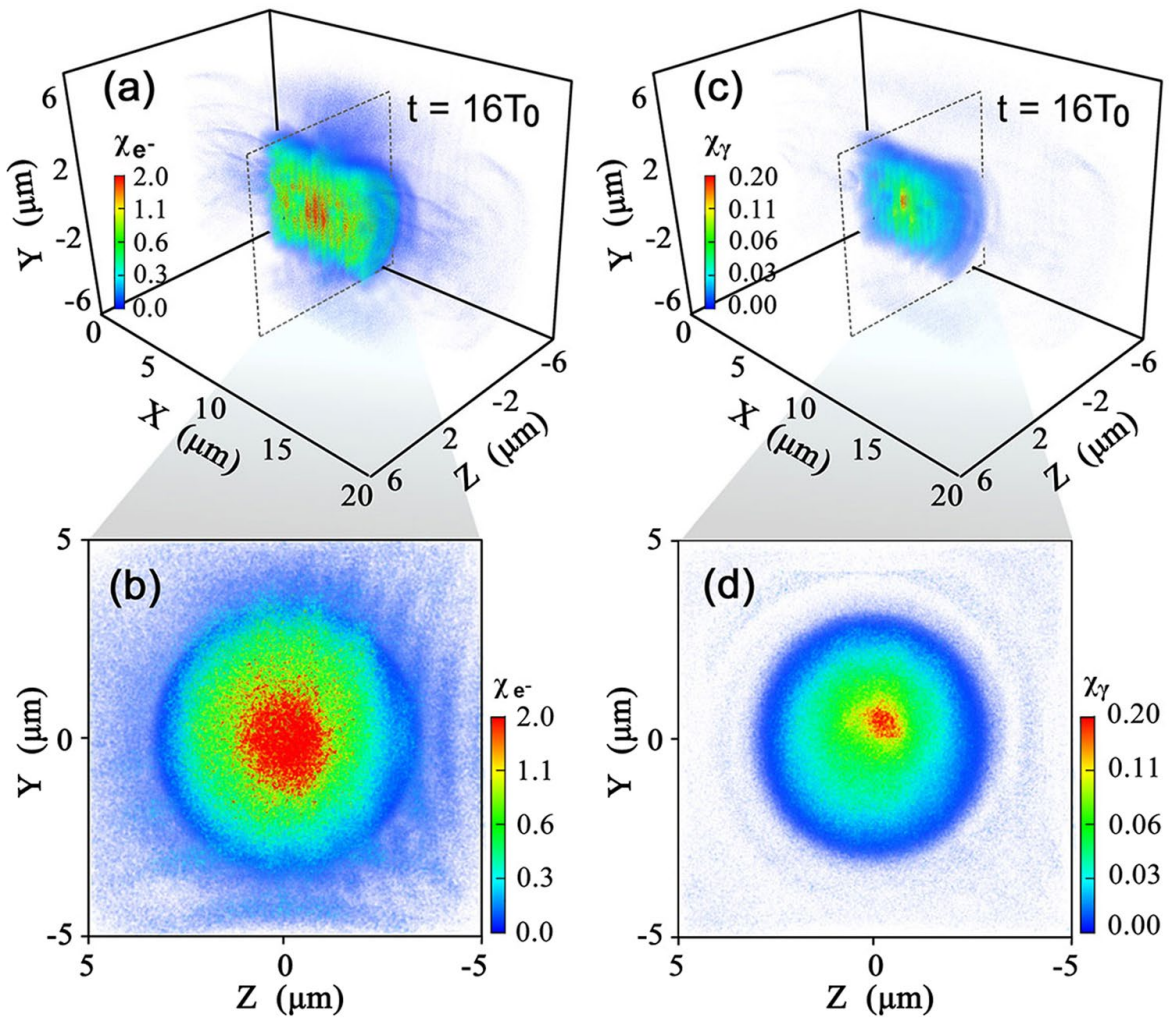
Numerically calculated quantum parameters ${\chi }_{{e}^{-}}$ (**a**) and ${\chi }_{\gamma }$ (**c**) at *t* = 16*T*_0_. (**b**) and (**d**) present the corresponding transverse distributions of both parameters at *x* = 10 *μm*.

**Figure 5:**
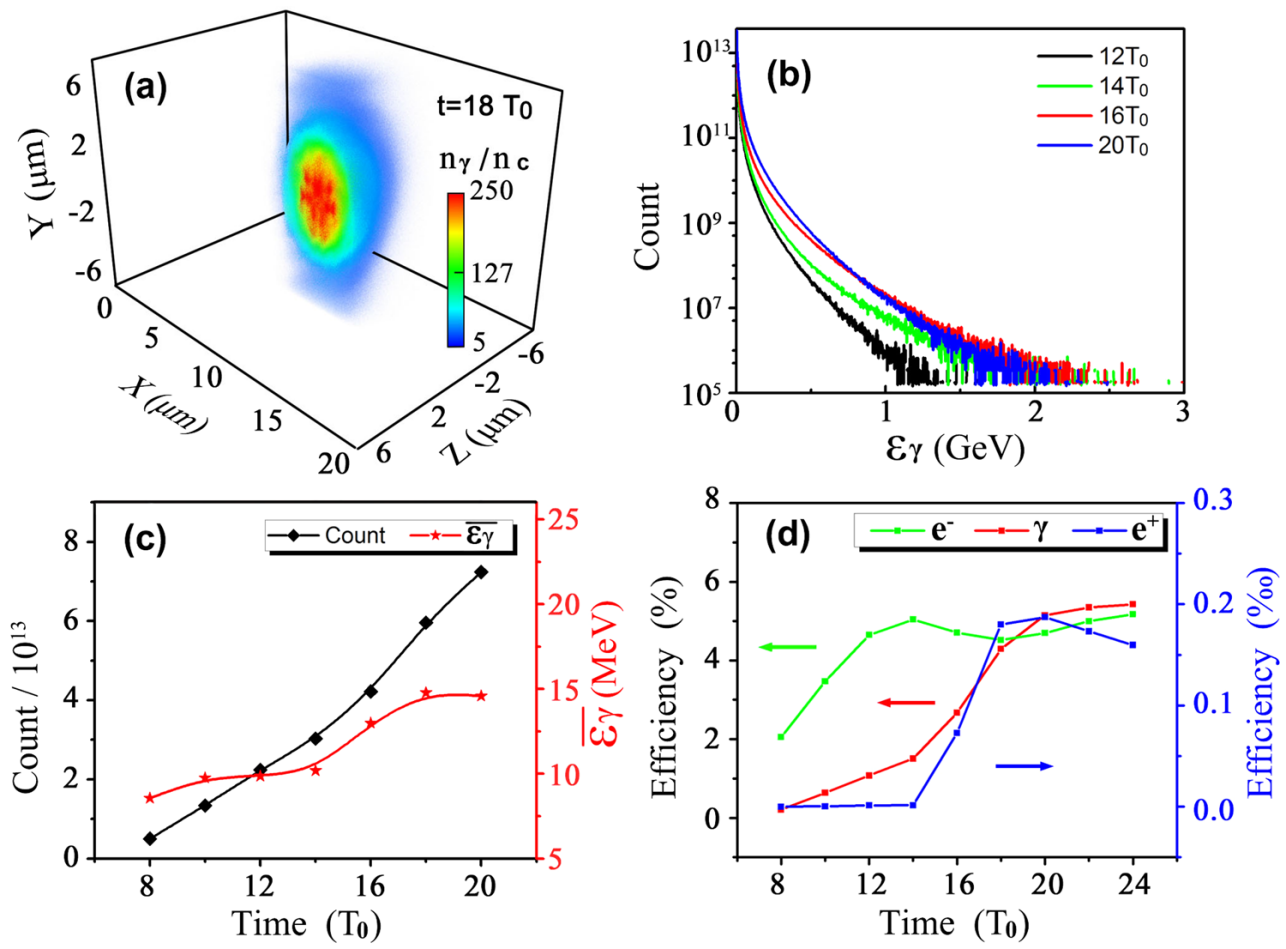
Density distribution of *γ*-photons at *t* = 18*T*_0_ (**a**) and energy spectrum of *γ*-photons (**b**). Evolution of the total number of *γ*-photons and their mean energy $\overline{{\varepsilon }_{\gamma }}$ (**c**). Laser energy conversion efficiency to electrons, positrons, and *γ*-photons (**d**). In (**c**) and (**d**) the points refer to the simulation results and the curves serve as guides to the eye.

Figure 4 illustrates the numerically calculated two key quantum parameters. Here the maximal value of ${\chi }_{{e}^{-},max} \sim 2$ at *t* = 16*T*_0_ and it gets much smaller on the lateral side of the deformed foils. The large ${\chi }_{{e}^{-}}$ results from the symmetric configuration in our scheme, which increases the equivalent quantum invariants of both ${\chi }_{{e}^{-}}$ and *χ*_*γ*_. Finally, QED effects are triggered and a large number of *γ*-photons are emitted with the maximal photon density up to 250*n*_*c*_ at *t* = 18*T*_0_ as seen in Fig. 5(a). Figure 5(b) presents the energy spectrum evolution of the scattered photons. The spectrum has a wide distribution ranging from MeV to several GeV. The average $\overline{{\chi }_{{e}^{-}}}$ can be approximated theoretically by $\mathrm{2(}\hslash {\omega }_{0}/{m}_{e}{c}^{2}){\overline{{a}_{f}}}^{2} \sim 0.5$, where $\overline{{a}_{f}}$ is the amplitude of mean electric fields. Thus the characteristic photon energy is written as^47^ $\hslash {\omega }_{\gamma }\simeq 0.44{\chi }_{{e}^{-}}{\overline{\gamma }}_{{e}^{-}}{m}_{e}{c}^{2} \sim 16.5$ MeV at 18*T*_0_, in excellent agreement with the simulation results in Fig. 5(c). Figure 5(d) presents the laser energy conversion efficiency to the electrons and secondary particles. We see the electron’s energy-share increases almost linearly until 13*T*_0_ as the foil transparency occurs, and then starts to decrease. This can be attributed to the abundant *γ*-photon emission on both sides. As time progresses, the photon number increases but the average photon energy remains after *t* = 18*T*_0_, since the high energy photons are continually consumed by the multi-photon BW process. The total laser-to-photon energy conversion efficiency is about 6%, which is comparable with that reported in a single foil^22^, though our laser intensity is only 1/4 of that. Note that this is a full 3D QED-PIC simulation and only *γ*-photons with energy larger than 1 MeV are counted.

The significant increase of the high energy *γ*-photons by comparing Fig. 2(g–h) can be attributed to Compton back-scattering of the transmitted laser photons from one side off the relativistic electrons from the other side. In this regime, nonlinear interactions in which an electron scatters simultaneously with two or more laser photons occurs. This is a highly-nonlinear process and prevails at ultra-high laser intensities. For a head-on collision, the maximum energy of scattered photons in a single-photon process^20^ is peaked at ${\varepsilon }_{\gamma ,max}=4{\gamma }_{{e}^{-}}^{2}\hslash {\omega }_{0}\mathrm{/(1}+4{\gamma }_{{e}^{-}}\hslash {\omega }_{0}/{m}_{e}{c}^{2})$, with average photon energy of $4{\gamma }_{{e}^{-}}^{2}\hslash {\omega }_{0}\mathrm{/3}$. Apparently, it is insufficient for an even 5 GeV electron to back-scatter the laser photons to such high photon energies in a single-photon process. Meanwhile, multiple inverse Compton scattering of scattered photons colliding with the energetic electrons is in principle possible, but the Compton cross section decreases by a factor 10^6^ ^33^, so that it fails to account for the huge number of *γ*-photon emission on both sides. This further proves that the high-order nonlinear Compton scattering dominates the photon emission in our scheme. Overall, the total *γ* photon number above 1 MeV is 7.23 × 10^13^ at *t* = 20*T*_0_ in such a *μm* × *μm* size with >6% laser energy conversion to the *γ*-photons. Conservatively, we take 2 × 10^13^ photons, a 1/*e* ^2^ source radius of 2*λ*_0_ × 2*λ*_0_, a divergence of 0.1 × 0.1 rad^2^, a bandwidth of 100%, and a pulse duration of about 5*T*_0_. This gives an unprecedented peak brightness of about 10^25^ photons/s/mm^2^/mrad^2^/0.1%BW at 15 MeV, which is four orders of magnitude higher than the current experiment record in laser-based Compton sources^18^ and 20-fold increase over that in the laser wakefield scheme with similar laser parameters^32^. The corresponding *γ*-rays intensity is 5 × 10^23^ Wcm^−2^ and the total emission power is 33 PW, almost 10^3^ times larger than reported in ref.^65^.

**Figure 6:**
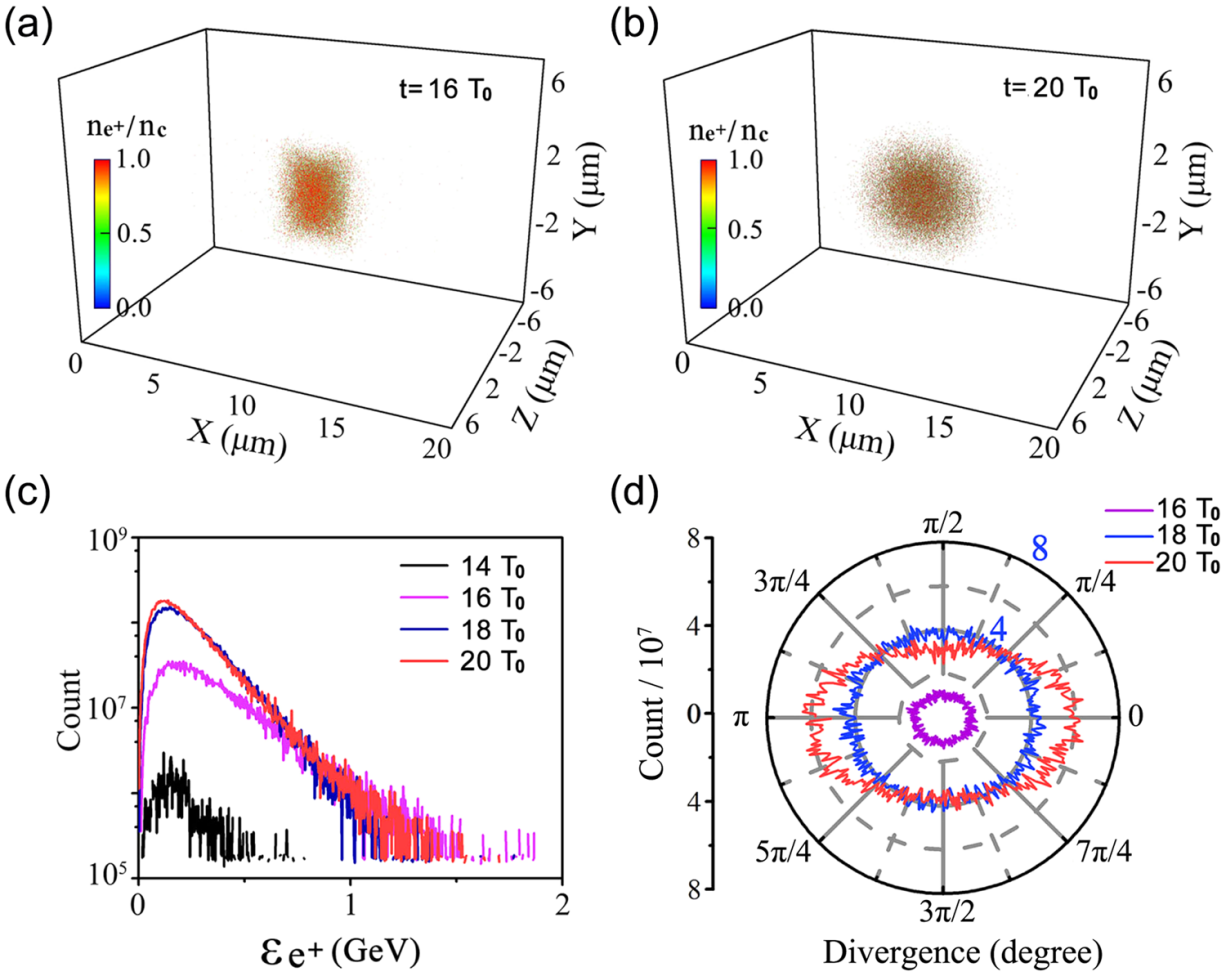
Distribution of the positron density at *t* = 16*T*_0_ (**a**) and 20*T*_0_ (**b**). The density above 1*n*_*c*_ is marked as red and the maximum density is up to 25*n*_*c*_. Energy spectrum of the positrons (**c**) and their divergence angle distribution at given times (**d**).

The numerically calculated parameter *χ*_*γ*_ determining the multi-photon BW process is given in Fig. 4(b). Using the above $\overline{{\chi }_{{e}^{-}}}$, we rewrite the average value of *χ*_*γ*_ as $\overline{{\chi }_{\gamma }}=2\times 0.44\overline{{\chi }_{{e}^{-}}}(\hslash {\omega }_{0}/{m}_{e}{c}^{2}){\overline{{a}_{f}}}^{2} \sim 0.08$. Although it is far from the threshold for the QED-cascade, it is sufficient to trigger the multi-photon BW process. Figure 6(a) and (b) show the positron density distribution at *t* = 16*T*_0_ and 20*T*_0_. The mean positron density achieved is about 4*n*_*c*_ with the maximal density up to 25 *n*_*c*_ along the laser axis at 20*T*_0_. This may provide us with an unique opportunity to investigate astrophysical phenomena in laboratories, which otherwise is impossible in the recent BH experiments^15,46^. Figure 6(c) presents the evolution of the positron energy spectrum. The positrons are continually accelerated by the laser pulse and their numbers increase significantly. However, these positrons also oscillate like electrons in the laser fields and emit *γ*-photons so that the number of high energy positrons decrease from 18*T*_0_ onwards. Meanwhile, the beam divergence decreases from 18*T*_0_ (see Fig. 6(d)) and most of the positrons are directed along the laser axis, indicating a good prospect for potential applications in future.

Our full 3D simulations show that the final positron number is 1.6 × 10^10^ at 20*T*_0_, which is much larger than that in the laser wakefield scheme^6,21,32^. The average positron energy is as high as 230 MeV. Assuming the volume of the focal area where *e* ^−^ *e* ^+^ pairs are created to be ~(*λ*/2)^3^, we may estimate the maximal positron flux by^8,29^.

4 $${N}_{{e}^{+},max}=\alpha {(\frac{\sqrt{1+{{\rm{\Xi }}}^{2}}{E}_{y}}{\alpha {E}_{S}})}^{\frac{5}{4}}{(\frac{{m}_{e}{c}^{2}}{\hslash {\omega }_{0}})}^{\frac{5}{2}},$$

where $\alpha ={e}^{2}/\hslash c$ is the fine-structure constant. Taking the peak electric field *E* ~ 285*E*_0_, we get ${N}_{{e}^{+},max}\approx 4.5\times {10}^{10}$, which is on the same level as the simulation results above. The corresponding laser energy conversion efficiency from the lasers to positrons is about 0.02%, as shown in Fig. 5(d). Taking $\overline{{\gamma }_{{e}^{+}}}=500$ and $\overline{{n}_{{e}^{+}}}=4{n}_{c}$, the relativistically corrected skin depth of the produced *e* ^−^ *e* ^+^ pair plasma is *δ*_*pair*_ ~ 2*λ*_0_. Thus the ratio of the plasma transverse size to the skin depth, ${D}_{pair}/{\delta }_{pair}\approx 2$, so that the collective effects of the pair plasma may be also triggered, e.g., Debye Shielding and plasma oscillation, which in turn affect the electron and positron dynamics. Due to the small resolution of the pair plasma (${D}_{pair}\approx 2{\delta }_{pair}$), it is impossible to resolve the collective dynamics of the pair plasma. This is beyond the scope of this paper, but it is of significance for investigation of laboratory astrophysical phenomena in laboratories^9–11^.

**Figure 7:**
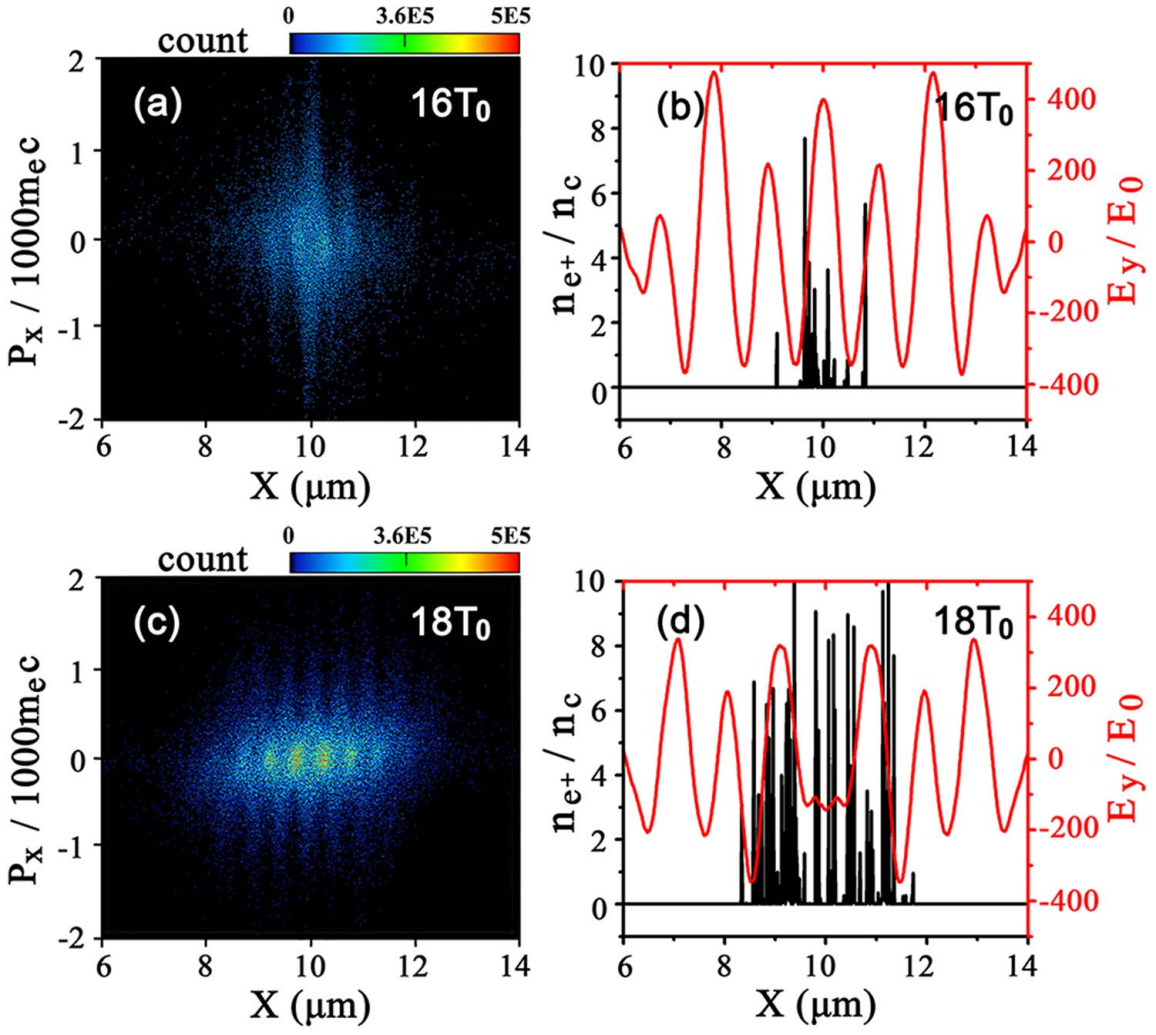
Phase space distribution (*x*,*p*_*x*_) of positrons at *t* = 16*T*_0_ (**a**) and 18*T*_0_ (**c**). The positron density distribution along the laser axis and the corresponding electric field *E*_*y*_ at *t* = 16*T*_0_ (**b**) and 18*T*_0_ (**d**).

The positrons obtained are well confined in a small size of the same order of the laser focal spot, which can be clearly seen from their phase space distributions exhibited in Fig. 7. This is credited to the standing waves formed directly by the colliding laser pulses, which help to trap the pairs at the nodes (*E* = 0) such as *x/λ*_0_ = 9.2,9.7,10.0,10.7, and prevent them from dispersing in space, so that the positrons can remain high energy and density until the laser pulses pass away. The wave structure with half laser wavelength spacing in Fig. 7(a) and (c) show the clear evidence of the positrons dynamics modulated by the standing waves.

## Discussion

There are several key factors to be considered for the experimental test and development of this concept. Firstly, the gap between the two foils plays an important role, because it determines the electron energy, density and flux. Since the carbon ion front moves with the proton layer, we can estimate the foil transparency time *τ* implicitly as

5 $$\begin{array}{c}G+2L > 2c\times {\int }_{0}^{\tau }\beta dt\\ \,\,\,\,\,\qquad =\,2c{\int }_{0}^{\tau }\mathrm{(1}-{[\nu (t)+\upsilon (t)]}^{\mathrm{1/3}}-{[\nu (t)-\upsilon (t)]}^{\mathrm{1/3}})dt\mathrm{.}\end{array}$$

In our simulations, the foils become completely transparent at about 15*T*_0_. Thus the critical foil gap is *G* > 2 × 6.94 − 2 × 0.32 = 12.88*λ*_0_ for the laser and foil parameters under discussion. This is in excellent agreement with the simulation results. Choosing a proper foil gap thus becomes a key issue in experiments. For example, a 10 *μm* gap is insufficient because the electrons have not yet been accelerated to high enough energies before the collision. In contrast, a 16 *μm* gap is so large that the transverse RTI develops significantly and the foil collapses more quickly before they collide. The resulting electron density and total number become lower, which is definitely undesirable for the *γ*-photon emission. Meanwhile, compared with a single foil^22–24^, which lacks flying foil’s dynamics at the initial stage and thus has smaller electron energies, we achieved more photons and positrons production due to the enhanced quantum parameters ${\chi }_{{e}^{-}}$ and *χ*_*γ*_. However, the spacing between the foils will also depend on the temporal profile of the laser pulses and an ultra-high laser contrast is required to facilitate the efficient RPA of DLC foils.

Secondly, we also find that the foil thickness and laser intensity play an important role in the foil dynamics and QED process. The laser intensity should be such that the foil can undergo a sufficient degree of acceleration and the transmitted light is still intense enough to penetrate the foil at the later stage. Considering the fact that *κ* depends on the foil thickness and laser intensity significantly, we can reduce the laser intensity and foil thickness simultaneously to keep *κ* unchanged. This leads to almost the same foil acceleration as above. For example, taking two 8 × 10^22^ Wcm^−2^ EP lasers with the same $\zeta =0.65$ and DLC foils with thickness of $L=0.25{\lambda }_{0}$, similar *γ*-photon emission and positron production can be achieved. The resulting brightness of the *γ*-rays and the maximum density of positrons are on the same order of magnitude. We have carried out a serials of 2D simulations to identify the optimal condition. It is finally shown that the optimal laser intensity should obey ${a}_{0}{n}_{c}/(\pi {n}_{e}) < L/{\lambda }_{0} < 2{a}_{0}{n}_{c}/(\pi {n}_{e})$ to enable highly efficient *γ*-photon emission and dense positron production.

**Figure 8:**
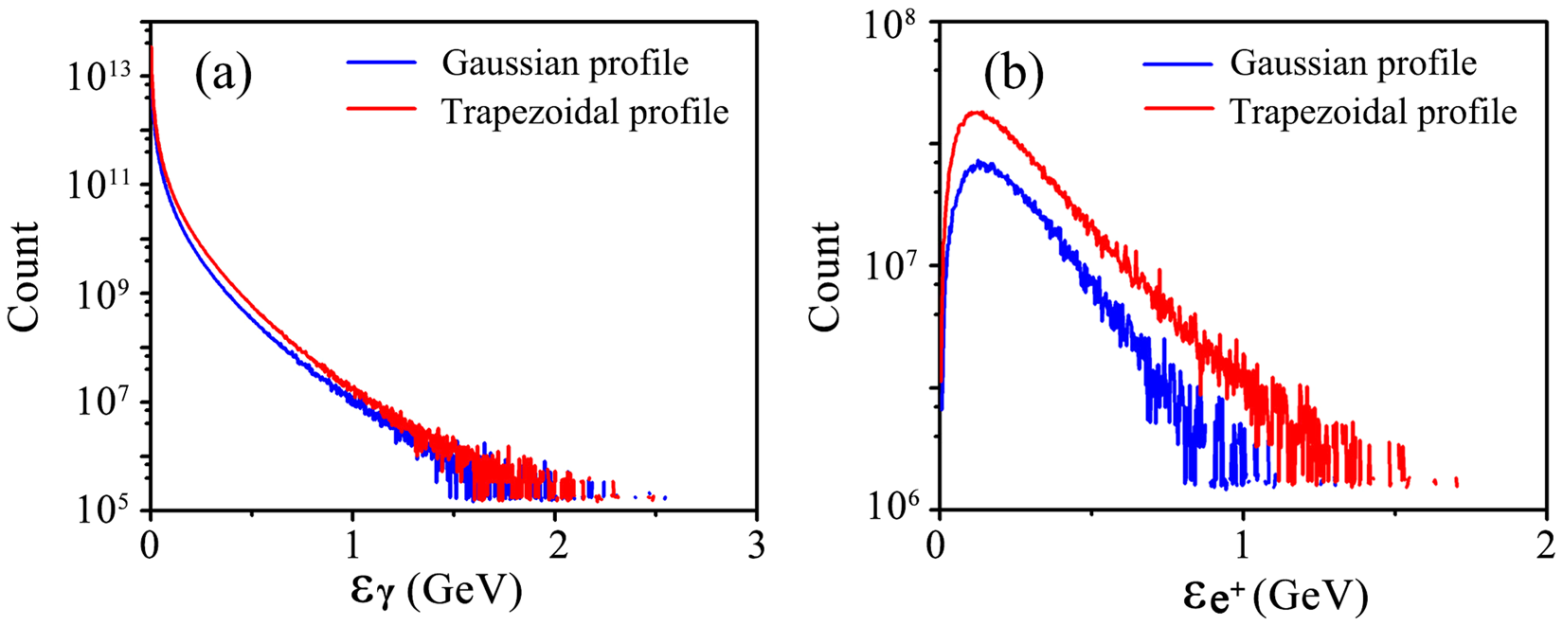
Energy spectrum of *γ*-photons and positrons in both cases at *t* = 20*T*_0_.

Thirdly, in order to check the influence of the laser temporal profile on the *γ*-photon emission and pair production, we considered a Gaussian time profile. In this case, the full-width-at-half-maximum (FWHM) of the laser pulse is *τ* = 6*T*_0_, to keep the total laser energy unchanged. The details of the laser parameters and simulation results are shown in Table 1 and Fig. 8. One can see that the magnitudes of the average energy, total number and energy spectrum of *γ*-photons in both cases are comparable. This indicates a weak dependence of *γ*-photon emission on the laser temporal profile in our scheme. Since the key parameter *χ*_*γ*_ is determined by the average laser intensity, the positron production will be more affected by use of a Gaussian time profile. The final positron number obtained is up to 0.6 × 10^10^, which is about 40% of that in the case with a trapezoidal profile. However, the average energies of positrons obtained are almost the same in both cases.

**Table 1:** Comparison of *γ*-photon emission and positron production in two cases with different laser temporal profiles at 20*T*_0_.

	Parameters	Trapezoidal profile case	Gaussian profile case
Laser	Time duration, *τ*_0_	(1-8-1)*T*_0_	6 *T*_0_
	Focal spot, *σ*_0_	4*λ*_0_	4*λ*_0_
	Peak intensity, *I*_0_	1.1 × 10^23^ Wcm^−2^	1.1 × 10^23^ Wcm^−2^
	Total energy, *ε*_*L*_	1580 J	1538.6 J
*γ*-photons	Average energy, $\overline{{\varepsilon }_{\gamma }}$	14.6 MeV	13.3 MeV
	Total number, *Nγ*	7.23 × 10^13^	4.80 × 10^13^
Positrons	Average energy, $\overline{{\varepsilon }_{{e}^{+}}}$	230 MeV	225 MeV
	Total number, ${N}_{{e}^{+}}$	1.61 × 10^10^	0.6 × 10^10^

In our configuration, bremsstrahlung is ignored since its cross section is highly dependent on the target atomic number^66,67^ and is proportional to $\alpha {r}_{e}^{2}{Z}^{2}\,\mathrm{ln}\,{\varepsilon }_{\gamma }$. Thus the cross section is significantly reduced by use of a low-Z target in our scheme. Additional simulations with the bremsstrahlung considered show that both the photon energy and numbers resulting from the bremsstrahlung is 10^5^ times smaller than those from the nonlinear Compton scattering. In our simulations, the trident process is switched off and the BH process is also ignored because the yield of the BH is expected to scale as *Z* ^4^ ^46^. It is interesting to compare our results with those observed in recent BH experiments. We see the obtained positron density is six orders of magnitude higher than reported in BH experiments (~10^16^ cm^−3^)^15,46^ and 80 times higher than predicted in the laser wakefield scheme^32^. The laser-to-positron energy conversion efficiency is at the same level as that in the BH experiments^46^ and much larger than in the laser wakefild scheme^32^.

Since the laser-driven DLC for ion acceleration has been demonstrated in recent experiments^36^. So the main issue for our scheme in future experiments would be the co-aligning of the two laser beams as well as their synchronization. Techniques have been developed for the co-aligning of two laser beams^68^. In addition, there is active work to synchronize high power laser beams to better than 10 fs (approaching femtosecond level). The two beams of 0.5 PW of Astra-Gemini laser can be synchronized to better than ±50 fs at present and this value will be improved in the near future^69^. The femtosecond synchronization of the two fs laser pulses can be obtained because both pulses are split from one fs pulse (after the laser oscillator), travel nearly identical optical paths (in the laser amplifier chains) and the small temporal differences are compensated at the end, before recombination. In additional simulations, we assume the left pulse irradiates the target normally but the right pulse is incident with 2 *μm* off the laser axis of the left pulse. All other parameters are the same as above. Finally, we find that the photon number obtained decreases from 5.96 × 10^13^ to 5.85 × 10^13^ (i.e., 2%) and the positron number also decreases from 1.45 × 10^10^ to 1.27 × 10^10^ (i.e., 14%) at *t* = 18*T*_0_. We therefore see a small reduction in the signal if the alignment is off by about the size of a laser focal radius. This further demonstrates the robustness of the scheme.

In conclusion, we propose an all-optical scheme for ultra-bright *γ*-ray emission and dense *e* ^−^ *e* ^+^ pair production by irradiating 10^22–23^ Wcm^−2^ colliding lasers onto two DLC foils which is investigated using full 3D PIC simulations. The electrons in the focal region of one foil are rapidly accelerated by the laser radiation pressure, forming flying RELs, and collide with the other laser pulse which penetrates through the second foil from the other side due to relativistically induced transparency. By incorporating the moving foil’s dynamics in the scheme, this dual interaction arrangement enables efficient Compton back-scattering and results in ultra-bright *γ*-photon emission with an unprecedented brightness of ~10^25^ photons/s/mm^2^/mrad^2^/0.1%BW at 15 MeV and intensity of 5 × 10^23^ Wcm^−2^. Finally, a GeV positron beam with a maximum density of 2.5 × 10^22^ cm^−3^ and flux of 1.6 × 10^10^/shot is achieved and collective effects of the pair plasma may be triggered, offering a window on investigating laboratory astrophysics at PW laser facilities.

## Methods

### Numerical modeling

The open source QED-PIC code EPOCH is employed, which has incorporated the binary collision, *γ*-ray emission, radiation reaction, and pair production by both the trident process and multi-photon BW process. A Monte Carlo algorithm with quantum correction is implemented in the code for calculating the photon emission and pair production by assigning each electron, positron, and *γ*-photon an optical depth, at which the emission occurs when the optical depth reaches a derived value from a pseudo-random number between 0 and 1. Bremsstrahlung is not included in EPOCH and is thus ignored in our scheme. Meanwhile, the trident process is switched off and the BH is also ignored because of the use of low-Z ultra-thin DLC targets. More details about the physical modules and algorithms can be found in the refs^47,48^.

The simulation box size is *X* × *Y* × *Z* = 20*λ*_0_ × 14*λ*_0_ × 14*λ*_0_, sampled by 2000 × 560 × 560 cells with 8 particles in each cell. Here *λ*_0_ = *cT*_0_ = 1 *μm* is the laser wavelength and *T*_0_ = 3.3 fs is the laser cycle. The foil electron density is ${n}_{{e}^{-}}$ = 200 *n*_*c*_, mixed with 20% protons in number density. Both foils have a thickness of *L* = 0.32 *μm* and radius of *R* = 5 *μm*, and are placed symmetrically at a distance of *D* = 3 *μm* away from the left and right boundaries, as shown in Fig. 1. The foil gap is *G* = 13.36 *μm*. Two identical EP Gaussian laser pulses with intensity component of *a*_*y*_ = 237 and *a*_*z*_ = 154 and focal size of *σ*_0_ = 4 *μm* irradiate the two foils simultaneously. Here, the ellipiticity of the laser pulse is chosen according to the simulation result^43^, which is favorable for the ion acceleration by suppressing the transverse instabilities. For comparison to the 1D theory as discussed in ref.^8^, each pulse has a trapezoidal profile in time with a duration of 10*T*_0_ (1*T*_0_ − 8*T*_0_ − 1*T*_0_). Thus the total laser energy of each laser pulse is about 1600 J. Here a typical 3D simulation presented requires two-week runtime using 600 CPU-cores on TianHe High Performance Computers at NUDT.

### Data availability

Data associated with research published in this paper can be accessed at http://dx.doi.org/10.15129/5a84ecba-17bd-4750-971c-82593cf45d77.

## References

[1] Yanovsky V (2008). Ultra-high intensity- 300-TW laser at 0.1 Hz repetition rate. Opt. Express.

[2] ELI Extreme Light Infrastructure. http://www.eli-beams.eu.

[3] CILEX Centre Interdisciplinaire Lumière EXtrême. http://cilexsaclay.fr.

[4] Di Piazza A, Müller C, Hatsagortsyan KZ, Keitel CH (2012). Extremely high-intensity laser interactions with fundamental quantum systems. Rev. Mod. Phys..

[5] Mourou GA, Tajima T, Bulanov SV (2006). Optics in the relativistic regime. Rev. Mod. Phys..

[6] Blackburn TG, Ridgers CP, Kirk JG, Bell AR (2014). Quantum radiation reaction in laser-electron-beam collisions. Phys. Rev. Lett..

[7] Bell AR, Kirk JG (2008). Possibility of prolific pair production with high-power lasers. Phys. Rev. Lett..

[8] Fedotov AM, Narozhny NB, Mourou G, Korn G (2010). Possibility of prolific pair production with high-power lasers. Phys. Rev. Lett..

[9] Remington BA (2005). High energy density laboratory astrophysics. Plasma Phys. Controlled Fusion.

[10] Ruffini R, Vereshchagin G, Xue S-S (2010). Electron-positron pairs in physics and astrophysics: From heavy nuclei to black holes. Phys. Rep..

[11] Ni L, Kliem B, Lin J, Wu N (2015). Fast magnetic reconnection in the solar chromosphere mediated by the plasmoid instability. Astrophys. J..

[12] Gonzalez-Izquierdo B (2016). Optically controlled dense current structures driven by relativistic plasma aperture-induced diffraction. Nat. Phys..

[13] Breit G, Wheeler JA (1934). Collision of two light quanta. Phys. Rev..

[14] Pike OJ, Mackenroth F, Hill EG, Rose SJ (2014). A photon-photon collider in a vacuum hohlraum. Nat. Photon..

[15] Sarri G (2015). Generation of neutral and high-density electron-positron pair plasmas in the laboratory. Nat. Commun..

[16] Liang EP, Wilks SC, Tabak M (1998). Pair production by ultraintense lasers. Phys. Rev. Lett..

[17] Nakashima K (2002). Numerical study of pair creation by ultraintense lasers. Phys. Plasmas.

[18] Ta Phuoc K (2012). All-optical Compton gamma-ray source. Nat. Photonics.

[19] Burke DL (1997). Positron production in multiphoton light-by-light scattering. Phys. Rev. Lett..

[20] Liu J-J, Yu T-P, Yin Y, Zhu X-L, Shao F-Q (2016). All-optical bright *γ*-ray and dense positron source by laser driven plasmas-filled cone. Opt. Express.

[21] Liu J-X (2016). Enhanced electron-positron pair production by ultra intense laser irradiating a compound target. Plasma Phys. Control. Fusion.

[22] Ridgers CP (2012). Dense electron-positron plasmas and ultra-intense bursts of Gamma-rays from laser-irradiated solid. Phys. Rev. Lett..

[23] Luo W (2015). Dense electron-positron plasmas and gamma-ray bursts generation by counterpropagating quantum electrodynamics-strong laser interaction with solid targets. Phys. Plasmas.

[24] Chang HX (2015). Generation of overdense and high-energy electron-positron-pair plasmas by irradiation of a thin foil with two ultraintense lasers. Phys. Rev. E.

[25] Yu T-P, Pukhov A, Sheng Z-M, Liu F, Shvets G (2013). Bright betatronlike X rays from radiation pressure acceleration of a mass-limited foil target. Phys. Rev. Lett..

[26] Zhu X-L (2015). Enhanced electron trapping and *γ* ray emission by ultra-intense laser irradiating a near-critical-density plasma filled gold cone. New J. Phys..

[27] Ribeyre X (2016). Pair creation in collision of *γ*-ray beams produced with high-intensity lasers. Phys. Rev. E.

[28] Kostyukova I, Yu., Nerush EN (2016). Production and dynamics of positrons in ultrahigh intensity laser-foil interactions. Phys. Plasmas.

[29] Zhu X-L (2016). Dense GeV electron-positron pairs generated by lasers in near-critical-density plasmas. Nat. Commun..

[30] Li HZ (2017). Ultra-bright *γ*-ray flashes and dense attosecond positron bunches from two counter-propagating laser pulses irradiating a micro-wire target. Opt. Express.

[31] Kirk JG, Bell AR, Arka I (2009). Pair production in counter-propagating laser beams. Plasma Phys. Controlled Fusion.

[32] Lobet M, Davoine X, d’Humières E, Gremille L (2017). Generation of high-energy electron-positron pairs in the collision of a laser-accelerated electron beam with a multipetawatt laser. Phys. Rev. Accel. Beams.

[33] Drebot I (2017). Matter from light-light scattering via Breit-Wheeler events produced by two interacting Compton sources. Phys. Rev. Accel. Beams.

[34] Macchi A, Cattani F, Liseykina TV, Cornolti F (2005). Laser acceleration of ion bunches at the front surface of overdense plasmas. Phys. Rev. Lett..

[35] Yu TP (2009). Quasimonoenergetic proton beam from ultraintense-laser irradiation of a target with holed backside. Phys. Plasmas.

[36] Henig A (2009). Radiation-pressure acceleration of ion beams driven by circularly polarized laser pulses. Phys. Rev. Lett..

[37] Esirkepov T, Borghesi M, Bulanov SV, Mourou G, Tajima T (2004). Highly efficient relativistic-ion generation in the laser-piston regime. Phys. Rev. Lett..

[38] Robinson APL, Zepf M, Kar S, Evans RG, Bellei C (2008). Radiation pressure acceleration of thin foils with circularly polarized laser pulses. New J. Phys..

[39] Yu T-P, Pukhov A, Shvets G, Chen M (2010). Stable laser-driven proton beam acceleration from a two-ion-species ultrathin foil. Phys. Rev. Lett..

[40] Macchi A, Veghini S, Pegoraro F (2009). “Light Sail” acceleration reexamined. Phys. Rev. Lett..

[41] Zou DB (2015). Enhanced laser-radiation-pressure-driven proton acceleration by moving focusing electric-fields in a foil-in-cone target. Phys. Plasmas..

[42] Yu TP, Chen M, Pukhov A (2009). High quality GeV proton beams from a density-modulated foil target. Laser Part. Beams.

[43] Wu D (2012). Suppression of transverse ablative Rayleigh-Taylor-like instability in the hole-boring radiation pressure acceleration by using elliptically polarized laser pulses. Phys. Plasmas.

[44] Liechtenstein VK (1997). Preparation and evaluation of thin diamond-like carbon foils for heavy-ion tandem accelerators and time-of-flight spectrometers. Nucl. Instrum. Methods Phys. Res..

[45] Bethe H, Heitler W (1934). On the stopping of fast particles and on the creation of positive electrons. Proc. R. Soc. Lond. A.

[46] Chen H (2009). Relativistic positron creation using ultraintense short pulse lasers. Phys. Rev. Lett..

[47] Ridgers CP (2014). Modelling gamma-ray photon emission and pair production in high-intensity laser-matter interactions. J. Comput. Phys..

[48] Duclous R, Kirk JG, Bell AR (2011). Monte carlo calculations of pair production in high-intensity laser plasma interactions. Plasma Phys. Control. Fusion.

[49] Yu TP (2011). Simulations of stable compact proton beam acceleration from a two-ion-species ultrathin foil. Phys. Plasmas.

[50] Qiao B (2012). Dominance of Radiation Pressure in Ion Acceleration with Linearly Polarized Pulses at Intensities of 10^21^ Wcm^−2^. Phys. Rev. Lett..

[51] Pegoraro F, Bulanov SV (2007). Photon bubbles and ion acceleration in a plasma dominated by the radiation pressure of an electromagnetic pulse. Phys. Rev. Lett..

[52] Chen M, Kumar N, Pukhov A, Yu TP (2011). Stabilized radiation pressure dominated ion acceleration from surface modulated thin-foil targets. Phys. Plasmas.

[53] Wang WQ (2015). Numerical investigation of the transverse instability of the radiation-pressure-driven foil. Phys. Rev. E.

[54] Zhang X (2007). Efficient GeV ion generation by ultraintense circularly polarized laser pulse. Phys. Plasmas.

[55] Chen M, Pukhov A, Yu T-P, Sheng Z-M (2010). Radiation reaction effects on ion acceleration in laser foil interaction. Plasma Phys. Control. Fusion.

[56] Tamburini M, Pegoraro F, Di Piazza A, Keitel CH, Macchi A (2010). Radiation reaction effects on radiation pressure acceleration. New J. Phys..

[57] Lehmann G, Spatschek KH (2012). Phase-space contraction and attractors for ultrarelativistic electrons. Phys. Rev. E.

[58] Bulanov SV (2010). Unlimited Ion Acceleration by Radiation Pressure. Phys. Rev. Lett..

[59] Bulanov SV (2010). Unlimited energy gain in the laser-driven radiation pressure dominant acceleration of ions. Phys. Plasmas.

[60] Palaniyappan S (2012). Dynamics of relativistic transparency and optical shuttering in expanding overdense plasmas. Nat. Phys.

[61] Wang WP (2012). Dynamic study of a compressed electron layer during the hole-boring stage in a sharp-front laser interaction region. Phys. Rev. Accel. Beams.

[62] Cattani F, Kim A, Anderson D, Lisak M (2000). Threshold of induced transparency in the relativistic interaction of an electromagnetic wave with overdense plasmas. Phys. Rev. E.

[63] Siminos E, Grech M, Skupin S, Schlegel T, Tikhonchuk VT (2012). Effect of electron heating on self-induced transparency in relativistic-intensity laser-plasma interactions. Phys. Rev. E.

[64] Schwinger J (1951). On gauge invariance and vacuum polarization. Phys. Rev..

[65] Stark DJ, Toncian T, Arefiev AV (2016). Enhanced multi-MeV photon emission by a laser-driven electron beam in a self-generated magnetic Field. Phys. Rev. Lett..

[66] Tsai Y-S (1974). Pair production and bremsstrahlung of charged leptons. Rev. Mod. Phys..

[67] Wan F, Lv C, Jia M, Sang H, Xie B (2017). Photon emission by bremsstrahlung and nonlinear Compton scattering in the interaction of ultraintense laser with plasmas. Eur. Phys. J. D.

[68] Faure J (2006). Controlled injection and acceleration of electrons in plasma wakefields by colliding laser pulses. Nature.

[69] Corvan, D. J. *et al*. Femtosecond-scale synchronization of ultra-intense focused laser beams. Preprint at http://arxiv.org/abs/1409.4243 (2014).

